# Yeast Methylotrophy: Metabolism, Gene Regulation and Peroxisome Homeostasis

**DOI:** 10.1155/2011/101298

**Published:** 2011-07-07

**Authors:** Hiroya Yurimoto, Masahide Oku, Yasuyoshi Sakai

**Affiliations:** ^1^Division of Applied Life Sciences, Graduate School of Agriculture, Kyoto University, Kitashirakawa-Oiwake, Sakyo-ku, Kyoto 606-8502, Japan; ^2^CREST, Japan Science and Technology Agency, 5 Sanbancho, Chiyoda-ku, Tokyo 102-0075, Japan

## Abstract

Eukaryotic methylotrophs, which are able to obtain all the carbon and energy needed for growth from methanol, are restricted to a limited number of yeast species. When these yeasts are grown on methanol as the sole carbon and energy source, the enzymes involved in methanol metabolism are strongly induced, and the membrane-bound organelles, peroxisomes, which contain key enzymes of methanol metabolism, proliferate massively. These features have made methylotrophic yeasts attractive hosts for the production of heterologous proteins and useful model organisms for the study of peroxisome biogenesis and degradation. In this paper, we describe recent insights into the molecular basis of yeast methylotrophy.

## 1. Introduction

Reduced C_1_-compounds, such as methane and methanol, are relatively abundant in nature. Methylotrophs, that have the ability to utilize C_1_-compounds as the sole source of carbon and energy, also appear to be ubiquitous in nature. A diverse range of prokaryotes and eukaryotes can utilize C_1_-compounds for growth, and methylotrophs have a diverse range of metabolic pathways for assimilating and dissimilating C_1_-compounds [1–4]. Prokaryotic methylotrophs can utilize a variety of C_1_-compounds (e.g., methane, methanol, methylamine), while eukaryotic methylotrophs can only use methanol as a carbon source, and methylamine not as a carbon source but as a nitrogen source. The latter group of organisms is limited to a number of yeast genera including *Candida*, *Pichia*, and some genera that were recently separated from *Pichia*, that is, *Ogataea*, *Kuraishia*, and *Komagataella* [5]. 

 Since the first isolation in 1969 [6], methylotrophic yeasts have been studied intensively in terms of both physiological activities and potential applications. In the early 1970s, production of single cell protein (SCP) using methanol as a carbon source was studied intensively [7, 8]. These studies established a high-cell-density cultivation method although large-scale production of SCP from methanol was eventually found not to be economically feasible. The metabolic pathways involved in methanol assimilation and dissimilation and characterization of the enzymes have been described in *Hansenula polymorpha *(*Pichia angusta*) and *Candida boidinii * [9–12]. One major finding was the strong inducibility of these enzymes by methanol. A variety of genes encoding enzymes and proteins involved in methanol metabolism have since been cloned, and the regulation of methanol-inducible gene expression has been studied [13, 14]. Methylotrophic yeasts also have been used as model organisms for peroxisome biogenesis and degradation, because methylotrophic growth in yeasts is accompanied by the massive proliferation of peroxisomes, membrane-bound organelles that contain several methanol-metabolizing enzymes [15–17]. 

 Heterologous gene expression systems driven by strong methanol-inducible promoters have been developed in a number of methylotrophic yeast strains, including *P. pastoris*, *H. polymorpha*, *P. methanolica*, and *C. boidinii* [18–22]. Increasing industrial and academic use of these expression systems has led to the heterologous production of a large number of proteins including enzymes, antibodies, cytokines, plasma proteins, and hormones. Some of the advantages of these systems include (i) cheap synthetic salt-based media for growing the yeast, (ii) strong and tightly regulated promoters induced by methanol and repressed by glucose or ethanol, and (iii) the fact that the processes of protein folding, secretion, and other functions in these yeasts are similar in many respects to the same processes in higher eukaryotes. 

 Recently, much attention has been paid to methanol as an alternative carbon source to replace coal and petroleum [23, 24]. Industrially, methanol is prepared from “syn-gas” (CO and H_2_) or reductive conversion of atmospheric CO_2_ with H_2_. Syn-gas can be produced from an abundant natural resource, methane, which is a major component of natural gas and is also obtained from renewable biomass [25]. Because methanol is a cheap and nonfood substrate, it has become a promising feedstock for biotechnological and chemical processes. In natural environments, methanol is produced by oxidation of methane by methane-oxidizing bacteria and also by decomposition of plant pectins and lignins that contain methylester and methoxyl groups, respectively [26, 27]. Methanol is oxidized to CO_2_ by methylotrophic bacteria and yeasts. Thus, methylotrophs play indispensable roles in the global carbon cycle between methane and CO_2_ called “the methane cycle.” A thorough understanding of the molecular basis of methylotrophy is needed not only to better understand the global methane cycle but also to permit more efficient use of methanol as a renewable carbon source.

## 2. Outline of Yeast Methanol Metabolism and the Physiological Roles of Associated Enzymes

**Figure 1:**
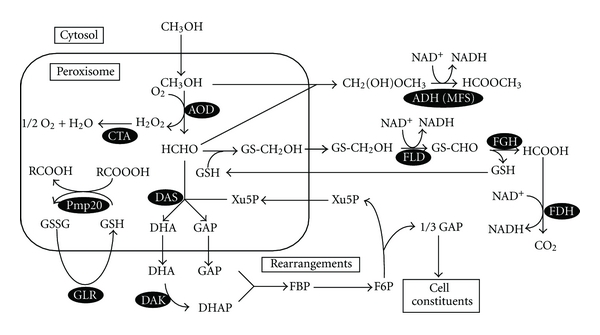
Outline of methanol metabolism in methylotrophic yeasts. *Enzymes: *ADH (MFS): alcohol dehydrogenase (methylformate-synthesizing enzyme); AOD: alcohol oxidase; CTA: catalase; DAK: dihydroxyacetone kinase; DAS: dihydroxyacetone synthase; FDH: formate dehydrogenase; FGH: *S*-formylglutathione hydrolase; FLD: formaldehyde dehydrogenase; GLR: glutathione reductase; Pmp20: peroxisome membrane protein which has glutathione peroxidase activity. *Abbreviations*: DHA: dihydroxyacetone; DHAP: dihydroxyacetone phosphate; F6P: fructose 6-phosphate; FBP: fructose 1,6-bisphosphate; GAP: glyceraldehyde 3-phosphate; GS-CH_2_OH: *S*-hydroxymethyl glutathione; GS-CHO: *S*-formylglutathione; GSH: reduced form of glutathione; GSSG: oxidized form of glutathione; RCOOOH: alkyl hydroperoxide; Xu5P: xylulose 5-phosphate.

 All methylotrophic yeasts use a common methanol-utilizing pathway. An outline of methanol metabolism in methylotrophic yeasts is summarized in Figure 1 [13]. Methanol is first oxidized by alcohol oxidase (AOD) to form formaldehyde and hydrogen peroxide, which are both highly toxic compounds. Formaldehyde is a central intermediate situated at the branch point between assimilation and dissimilation pathways [2]. A portion of formaldehyde is fixed to xylulose 5-phosphate (Xu5P) by dihydroxyacetone synthase (DAS) forming dihydroxyacetone (DHA) and glyceraldehyde 3-phosphate (GAP), which are used for the synthesis of cell constituents and the regeneration of Xu5P. AOD and DAS are located in peroxisomes together with catalase (CTA), which breaks down hydrogen peroxide. DHA and GAP are further assimilated within the cytosol. DHA is phosphorylated by dihydroxyacetone kinase (DHAK), and, subsequently, dihydroxyacetone phosphate (DHAP) and GAP form fructose 1,6-bisphosphate which is then utilized for regeneration of Xu5P and for biosynthesis of cell constituents. 

 Another portion of formaldehyde is further oxidized to CO_2_ by the cytosolic dissimilation pathway. Formaldehyde generated by AOD reacts nonenzymatically with the reduced form of glutathione (GSH) to generate *S*-hydroxymethyl glutathione (*S*-HMG). *S*-HMG is then oxidized to CO_2_ through the cytosolic GSH-dependent oxidation pathway, which is ubiquitous in nature [36]. NAD^+^-linked and GSH-dependent formaldehyde dehydrogenase (FLD) uses *S*-HMG as a substrate to yield *S*-formylglutathione (*S*-FG) and NADH. *S*-FG is then hydrolyzed to formate and GSH by *S*-formylglutathione hydrolase (FGH). NAD^+^-linked formate dehydrogenase (FDH) is the last enzyme involved in the methanol dissimilation pathway and generates CO_2_ and NADH through the oxidation of formate. 

 We have studied the physiological role of the methanol-metabolizing enzymes by cloning and disrupting the corresponding genes in *C. boidinii * [22, 37]. The cloned genes and phenotypes of the associated mutants are summarized in Table 1. Obviously, AOD and DAS are both essential enzymes for methylotrophy in yeast [28, 29]. However, the enzymes involved in the GSH-dependent formaldehyde oxidation pathway have different roles in methanol metabolism. The FLD1-disrupted strain (*fgh1Δ*) was unable to grow on methanol under chemostat conditions even with a low dilution rate, indicating that FLD is essential for growth on methanol [30]. The *FGH1*- and *FDH1*-disrupted strains (*fgh1Δ* and *fdh1Δ*, resp.), however, were able to grow on methanol as a sole source of carbon and energy, although the growth yields were only 10% and 25% of that observed for the wild-type strain, respectively [31, 32]. These results suggested that although FGH and FDH are not essential, they contribute to optimal growth in the presence of methanol. We further showed that methyl formate synthesis catalyzed by cytosolic alcohol dehydrogenase (ADH) contributed to formaldehyde detoxification through GSH-independent formaldehyde oxidation during growth on methanol [33].

## 3. Roles and Function of Peroxisomes in Methanol Metabolism

 Oxidation of methanol in methylotrophic yeasts results in the formation of two very reactive and toxic compounds, formaldehyde and hydrogen peroxide. The enzymes involved in the metabolism of these compounds, namely AOD, DAS, CTA, and Pmp20, are compartmentalized in peroxisomes [16]. The presence of hydrogen peroxide-producing oxidases and catalase in a shared compartment is a characteristic feature of peroxisomes in all eukaryotes which offers the advantage of tightly linking production and breakdown of this toxic compound, preventing its diffusion into the cytosol [15]. The physiological roles of peroxisomal antioxidant enzymes and the antioxidative responses underlying yeast methylotrophy are described below (see Section 5). 

 Both AOD and DAS are major components of the peroxisomal matrix, suggesting that the generation and fixation of formaldehyde is primarily confined to this organelle. Compartmentalization of the formaldehyde conversion reactions in a membrane-bound organelle may be one cellular strategy to avoid formaldehyde toxicity. Furthermore, GSH was also shown to be present in the peroxisome at a physiologically significant level [35], indicating that *S*-HMG can be formed within peroxisomes and then exported to the cytosol [30, 31].

 The peroxisomal localization of peroxisome matrix enzymes is essential for allowing methylotrophic yeasts to grow on methanol [38, 39]. Intact peroxisomes are crucial to support growth of cells on methanol as a sole source of carbon and energy. The function of peroxisomes during methylotrophic growth is (i) to allow proper partitioning of formaldehyde generated from methanol via the assimilation and dissimilation pathways and (ii) to provide a site for scavenging hydrogen peroxide. These characteristics have been an important tool in the isolation of peroxisome-deficient mutants (*pex*) of methylotrophic yeast species, as such mutants lose the capacity to grow on methanol despite the fact that they have all the enzymes needed for methanol metabolism [40, 41]. Detailed physiological studies of yeast *pex* mutants have revealed why compartmentalization is essential for methylotrophic growth [42]. Many *PEX* genes encoding peroxins, which are proteins involved in peroxisome biogenesis, peroxisomal matrix protein import, membrane biogenesis, peroxisome proliferation, and peroxisome inheritance, have been identified and later characterized using methylotrophic yeasts as model organisms [16].

## 4. Regulation of Methanol-Inducible Gene Expression in Methylotrophic Yeast

 The key enzymes of methanol metabolism mentioned above are highly induced by methanol and are virtually absent in cells growing on glucose. We have studied the regulation of the expression of genes encoding these enzymes in cells grown on various carbon and nitrogen sources in *C. boidinii*. Furthermore, we have evaluated in detail the strength and regulation of the methanol-inducible promoters using a sensitive reporter system based on the acid phosphatase gene (*PHO5*) from *Saccharomyces cerevisiae* [43]. 

**Figure 2:**
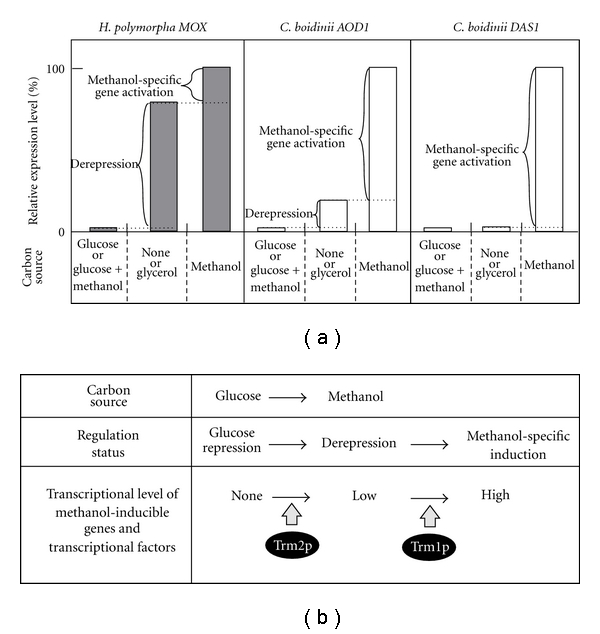
Molecular mechanism of methanol-inducible gene expression. (a) Relative expression levels of *H. polymorpha MOX* (encoding AOD), *C. boidinii AOD1*, and *C. boidinii DAS1* during growth on various carbon sources. On glucose-containing media, expression is completely repressed. When glucose is completely consumed or cells are shifted to glycerol medium, a derepressed level of expression of the AOD genes is induced (derepression) and the extent of derepression of the AOD genes differs between *H. polymorpha* and *C. boidinii*. When cells are grown on methanol, the maximum level of expression of AOD genes is achieved not only by derepression but also by methanol-specific gene activation. The induction of *DAS1* on methanol medium is achieved only by methanol-specific gene activation. (b) During growth on glucose, expression of methanol-inducible genes is repressed. When cells are shifted to methanol, initially, a Trm2p-related derepression event occurs followed by a Trm1p-related methanol-specific gene activation.

 Maximal expression of the methanol-inducible genes is thought to be exerted through two modes of regulation, that is, derepression and methanol-specific gene activation [14, 22, 37]. The former refers to activation of genes in a manner independent of the availability of methanol, and the latter refers to activation of genes in response to methanol (Figure 2(a)). When grown on glycerol, *C. boidinii* and *H. polymorpha *exhibited ~10 and 80% of the methanol-induced maximum AOD expression level, respectively. Glucose-limited chemostat culture experiments also showed that the levels of AOD in *H. polymorpha* gradually increased with decreasing dilution, whereas the derepression of AOD was lower in *C. boidinii* than in *H. polymorpha *[44]. Therefore, the extent and mode of derepression differs among methylotrophic yeast species. In* C. boidinii*, the *AOD1* promoter was shown to have a maximum level of expression in cells grown on methanol, a derepressed level of expression in cells grown on glycerol or oleate, and was repressed in cells grown on glucose or ethanol (Figure 2(a)). The *DAS1 *promoter did not appear to be derepressed when cells were grown on any of the alternative carbon sources [43], indicating that the derepressed level of expression of methanol-inducible genes is gene-specific even in *C. boidinii*. Based on these observations, we were able to determine a number of genetic factors specific to methanol induction in *C. boidinii*, particularly in regard to methanol-specific gene activation as opposed to derepression. 

 In *H. polymorpha*, Mpp1p regulates levels of peroxisomal matrix proteins and peroxins [45]. Further, the SWI/SNF complex (Swi1p and Snf2p) in *H. polymorpha *plays a role in the transcriptional control of methanol-inducible gene expression, suggesting that chromatin remodeling participates in the transcriptional regulation of methanol-inducible genes [46]. In *P. pastoris*, Mxr1p (a homologue of the C_2_H_2_-type transcriptional factor Adr1p in *S. cerevisiae*) was shown to control transcription of genes involved in methanol metabolism, in particular *AOD1*, as well as the *PEX *genes [47]. All of these transcriptional regulators appear to be involved in glucose derepression of gene expression; however, we have also identified a novel gene, *TRM1*, as a putative regulator of methanol-specific gene activation [48].

 Trm1p belongs to the Zn(II)_2_Cys_6_-type zinc cluster protein family, members of which are known to be transcriptional regulators in fungi [49]. Deletion of the *TRM1 *gene completely prevented growth on methanol, whereas it did not cause a defect in growth on glucose or other nonfermentative carbon sources, including glycerol, ethanol, or oleic acid. These results suggested that Trm1p is more involved in the regulation of methanol-specific gene activation than in derepression. The transcriptional activities of all of the methanol-inducible promoters tested drastically decreased in the *trm1Δ* strain grown in methanol medium. Thus, Trm1p was shown to be a master transcriptional regulator of methanol-specific gene activation in the methylotrophic yeast *C. boidinii*. 

 With respect to derepression, we recently isolated and characterized Trm2p, which is homologous to *P. pastoris *Mxr1p and *S. cerevisiae* Adr1p [50]. A *C. boidinii* mutant (*trm2Δ*) could not grow on methanol or oleate but could grow on glucose or ethanol. Trm2p was necessary for the activation of five methanol-inducible promoters tested. The derepressed level of expression of *AOD1, *which was observed in the *trm1Δ* strain, decreased in the *trm1Δ trm2Δ* strain to a level similar to that observed in the *trm2Δ* strain. These results suggest that Trm2p-dependent derepression is essential for Trm1p-dependent methanol-specific gene activation in *C. boidinii* (Figure 2(b)). 

 Recently, a transcriptome analysis of *H. polymorpha *cells shifted from glucose- to methanol-containing media was reported [51]. As expected, genes involved in methanol metabolism and several *PEX *genes were highly upregulated after 2 hours incubation on methanol. Among 1184 genes, which were significantly upregulated with at least a two-fold expression, highest upregulation (>300-fold) was observed for the genes encoding a dissimilation pathway enzyme FDH and a transcriptional factor Mpp1. Autophagy-related genes (*ATG *genes; see Section 6) were also upregulated. In *P. pastoris*, autophagy was shown to be induced at the lag phase of the methylotrophic growth as described below [52]. 

## 5. Antioxidative Responses Underlying Methylotrophy in Yeasts

 Because methanol metabolism in methylotrophic yeasts inevitably produces hydrogen peroxide, the organisms possess functions protective against oxidative stress, including induction of CTA and glutathione peroxidases. In *C. boidinii*, a CTA-deficient mutant (*cta1Δ*) was able to grow on methanol as a sole carbon source although its growth rate was much lower than that of the wild-type strain. The peroxisomal localization of CTA is essential for its function [34]. A 20-kDa peroxisomal peripheral membrane protein of *C. boidinii* (Pmp20) was identified as peroxiredoxin, an enzyme with antioxidant activity necessary for methylotrophic growth [35]. Interestingly, the *pmp20Δ* strain had a more severe growth defect than the* cta1Δ* strain. Pmp20 is localized to peroxisomes and has the ability to reduce alkyl hydroperoxide species, which suggests a role in elimination of other reactive oxygen species derived from hydrogen peroxide (Figure 1).

**Figure 3:**
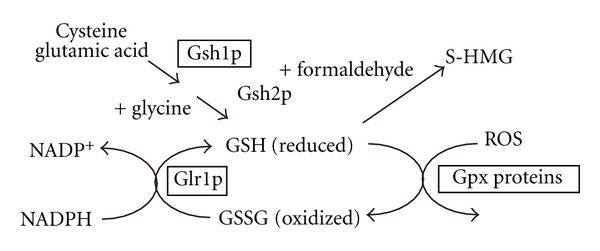
Schematic drawing of GSH dynamics and its regulation by Yap1. The proteins enclosed within the boxes represent factors found to be induced by Yap1 at the level of transcription.* Enzymes: *Gsh1p: *γ*-glutamylcysteine synthetase; Gsh2p: glutathione synthetase; Glr1p: glutathione reducatse; Gpx: glutathione peroxidase.* S*-HMG: *S*-hydroxymethyl glutathione; GSH: reduced form of glutathione; GSSG: oxidized form of glutathione; ROS: reactive oxygen species.

 In order for the system that protects cells against oxidative stress to function properly, sufficient GSH is necessary to serve as a cosubstrate in the reaction mediated by glutathione peroxidases (Figure 3). GSH is also utilized to form *S*-HMG, a formaldehyde conjugate that serves as an intermediate metabolite in the catabolism of methanol. Thus, it is plausible that the amount of GSH available for catabolic and antioxidative pathways plays a vital role in yeast methylotrophy. 

 Upregulation of GSH is accomplished through induction of its *de novo* synthesis and regeneration. The synthesis pathway begins with the reaction catalyzed by Gsh1p (*γ*-glutamylcysteine synthetase) to form *γ*-glutamylcysteine. The second and last step catalyzed by Gsh2p (glutathione synthetase) conjugates L-glycine to *γ*-glutamylcysteine. Because the Gsh1p-mediated reaction is rate limiting, induction of Gsh1p expression plays a central role in upregulating GSH levels. The regeneration of free GSH from its oxidized form is catalyzed by Glr1p (glutathione reductase) at the expense of NADPH. In addition, the GSH pool is also partially replenished from its conjugate form *S*-HMG, via the activities of FLD and FGH. Thus, we anticipated that deciphering the molecular mechanisms governing induction of these enzymes would be important for elucidating redox-related regulatory mechanisms in methylotrophic yeasts. 

 A recent study demonstrated that a transcription factor termed Yap1 plays an important role in the upregulation of GSH-related enzymes in *P. pastoris* [53]. Disruption of *PpYAP1* led to defects in transcriptional induction of genes encoding PpGlr1p, PpGsh1p, and a glutathione peroxidase PpGpx1p and caused severe growth arrest in methanol culture. PpYap1p fused to a fluorescent tag was found to change localization from the cytoplasm to the nucleus several hours after the onset of methanol culture, validating the role of PpYap1 in the antioxidative response. Loss of genes required for GSH regeneration from *S*-HMG (*PpFLD1* or *PpFGH1*) enhanced Yap1 translocation, which suggests an increased demand for GSH in these gene mutants. Of note, disruption of *PpGLR1* caused a severe growth defect concomitantly with abnormal accumulation of formaldehyde in methanol culture, showing that regeneration of GSH from its oxidized form plays a vital role in methylotrophy in this organism. 

 The molecular details of PpYap1p response were further investigated in another study [54]. PpYap1p has two cysteine-rich domains (CRDs) similar to its homologue in* S. cerevisiae*. Each of the CRDs has two cysteine residues essential for induction of *TRR1* (encoding thioredoxin reductase) in response to hydrogen peroxide treatment. This response was dependent on PpGpx1p, similar to the *S. cerevisiae* Yap1p response that requires ScGpx3p. These functional characteristics of PpYap1 are thought to underlie antioxidant activities in methanol culture.

## 6. Autophagic Activities for Regulations of Methylotrophy

 As mentioned above, one remarkable feature of methylotrophy in eukaryotes is that many of the enzymes required for methanol metabolism are located in peroxisomes. This means that the regulation of methanol metabolism can be accomplished in part, through control of organelle homeostasis. Methylotrophic yeasts have been utilized as valuable experimental systems for elucidating molecular mechanisms of peroxisome biogenesis and degradation [55]. Studies in these yeasts in fact contributed to establishing terms now widely used for genes involved in peroxisome biogenesis, the so-called *PEX* genes, and autophagy-related genes (*ATG*). Below, we briefly summarize what is known about the involvement of autophagy in regulating peroxisome homeostasis. 

 The term autophagy refers to the transport of cytoplasmic components to the lysosome/vacuole, followed by their degradation inside the lysosome/vacuole. Methylotrophic yeasts have peroxisome-selective autophagic machinery to reduce peroxisome quantity. This activity, termed pexophagy, is induced when nutrient conditions change from those requiring peroxisome function (e.g., growth in methanol culture) to those irrelevant to peroxisomal activities (e.g., growth in glucose or ethanol culture) [56, 57]. 

**Figure 4:**
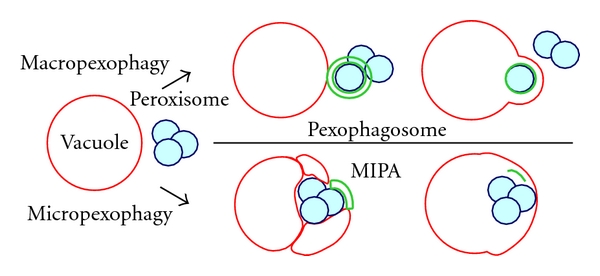
Model of organelle dynamics observed during pexophagy in methylotrophic yeasts. The green portions of the figure (MIPA and pexophagosome) represent autophagic membrane structures newly synthesized by the action of Atg proteins.

 Pexophagy has been classified into two modes according to differences in organelle dynamics (Figure 4) [58]. In macropexophagy, the peroxisome is encapsulated within a newly synthesized double-membrane structure termed the pexophagosome. After sequestering the peroxisome from the cytoplasm, the pexophagosome fuses with the limiting membrane of the vacuole, resulting in release of its content and its inner membrane into the vacuole. In micropexophagy, a portion of the peroxisome surface is covered by a flat double-membrane structure termed the micropexophagy apparatus, or MIPA [59]. The remainder of the peroxisome surface is engulfed by extension of the vacuolar membrane, with fusion between the MIPA and the vacuolar membrane sealing the peroxisome within the vacuolar compartment. To date, micropexophagy has only been observed in* P. pastoris*. 

 Most proteins responsible for pexophagy are also needed for other autophagic pathways and are now considered Atg proteins, as noted above. This is because in many autophagic pathways, including the two modes of pexophagy, *de novo *synthesis of membrane structures plays a crucial role, requiring a shared molecular machinery for modulating membrane dynamics. Studies to elucidate the molecular mechanisms by which Atg proteins form membrane structures have been a major focus of research efforts [60].

 A recent study demonstrated that autophagic activity also plays an important role in peroxisome biogenesis in methylotrophic yeast [52]. Autophagy was reported to be induced at lag phase following a shift from growth on glucose to methanol. This induction was observed only in minimal medium as addition of excess amino acids inhibits the induction. Loss of several *ATG *genes led to a prolonged lag phase, indicating a physiological function of for autophagic activity during adaptation to growth on methanol. The carbon source shift forces cells to remodel metabolic systems and to increase the peroxisome quantity, which requires a substantial supply of amino acids. The induced autophagy (termed lag-phase autophagy) should have the effect of increasing amino acid pools through degradation of cellular components in the vacuole.

## 7. Perspectives

 Heterologous gene expression systems using methylotrophic yeasts are based on the unique methanol metabolism of these organisms and are characterized by strong and tightly regulated methanol-inducible gene expression We anticipate that additional studies of the molecular basis for methylotrophy in yeasts that focus on transcriptional machinery and signal transduction pathways involved in methanol-inducible gene expression, and mechanisms that regulate organelle homeostasis and redox state will lead to improvements in these heterologous gene expression systems.

 Methylotrophs play important roles in carbon recycling in natural environments. The symbiotic relationship between plants and methylotrophic bacteria has been the focus of recent intensive study [61–63]. In contrast, the relationship between plants and methylotrophic yeasts has not been extensively characterized, although methylotrophic yeasts have often been isolated from plant-related materials [5, 16]. We previously reported that *C. boidinii *can grow on pectin and that this ability depends on methylotrophy [26]. Further intensive analysis of the molecular basis of yeast methylotrophy is expected to reveal new physiological functions and their importance in natural environments.

## Figures and Tables

**Table 1:** Phenotypes associated with disruptions in genes encoding methanol-metabolizing enzymes in *C. boidinii*.

Enzyme	Gene name	Mutant (M)/disruptant (D) phenotype	Reference
Alcohol oxidase	*AOD1*	no growth	[28]
Dihydroxyacetone synthase	*DAS1*	no growth	[29]
Formaldehyde dehydrogenase	*FLD1*	no growth	[30]
*S*-Formylglutathione hydrolase	*FGH1*	weak growth	[31]
Formate dehydrogenase	*FDH1*	weak growth	[32]
Alcohol dehydrogenase	*ADH1*	weak growth	[33]
Catalase	*CTA1*	weak growth	[34]
Pmp20	*PMP20*	no growth	[35]
